# CX3CR1 upregulation modulates microglial activation and preserves synapses in the hippocampus and frontal cortex of middle-aged mice

**DOI:** 10.3389/fragi.2025.1549848

**Published:** 2025-07-31

**Authors:** Jinfeng Liu, Zhongqing Sun, Xin Liu, Kin Chiu, Lan Ma, Jiantao Wang

**Affiliations:** ^1^ Shenzhen Eye Hospital, Jinan University, Shenzhen Eye Institute, Shenzhen, China; ^2^ Department of Neurology, Xijing Hospital, Fourth Military Medical University, Xi’an, China; ^3^ State Key Laboratory of Brain and Cognitive Sciences, The University of Hong Kong, Hong Kong, Hong Kong SAR, China; ^4^ Department of Psychology, The University of Hong Kong, Hong Kong, Hong Kong SAR, China; ^5^ Institute of Biopharmaceutical and Health Engineering, Tsinghua Shenzhen International Graduate School, Tsinghua University, Shenzhen, China; ^6^ State Key Laboratory of Chemical Oncogenomics, Tsinghua Shenzhen International Graduate School, Tsinghua University, Shenzhen, China; ^7^ Institute of Biomedical Health Technology and Engineering, Shenzhen Bay Laboratory, Shenzhen, China

**Keywords:** CX3CR1, microglia, aging brain, synaptic homeostasis, hippocampus, frontal cortex, neuroinflammation, mouse model

## Abstract

**Introduction:**

The aging brain shows alterations in microglial function, morphology, and phenotype, reflecting a state of chronic activation. CX3CR1 plays a critical role in regulating microglial chemotaxis, phagocytosis, and activation. However, its exact role in the aging brain is not well understood.

**Methods:**

In this study, we examined the expression of CX3CR1 in the brains of middle-aged mice (10 months old) and explored its functional implications by measuring cytokine and scavenger receptor expression, analyzing microglial and astrocyte morphology, conducting proteomic profiling, and assessing synapse density in CX3CR1-deficient mouse brain.

**Results:**

Our results showed that CX3CR1 was upregulated in the hippocampus and frontal cortex of middle-aged mice, with decreased IL-1α and IL-1β levels in the frontal cortex and increased SRA and RAGE levels in the hippocampus. Proteomic analysis revealed an enrichment of differentially expressed proteins (DE-proteins) in the cerebrum of middle-aged mice in GO pathways such as “synapse”, “translation”, and “ribosome”. Following CX3CR1 knockout in the middle-aged mice, TNF-α and IL-1α levels increased, while CD68, SRA, and RAGE levels decreased in the hippocampus. Similarly, CD68, CD36, SRB1, and RAGE levels decreased in the frontal cortex. The absence of CX3CR1 significantly altered microglial morphology, resulting in enlarged cell bodies and shortened processes in the hippocampus and frontal cortex. CX3CR1 deficiency also changed astrocyte morphology, leading to enlarged cell bodies and elongated processes in the hippocampus. Further proteomic analysis indicated that CX3CR1 deficiency affected protein levels in GO pathways such as “glutamatergic synapse” and “RNA splicing.” Additionally, we observed a reduction in synaptophysin-positive synapse density in both the hippocampus and frontal cortex of CX3CR1-deficient mice.

**Discussion:**

Our findings demonstrated that CX3CR1 was upregulated to maintain synaptic homeostasis probably through regulating microglial activation and phagocytosis in the brains of middle-aged mice. CX3CR1 may represent a promising therapeutic target for alleviating the effects of aging and preventing neurodegeneration.

## 1 Introduction

With the global increase in life expectancy over recent decades, the population aged 60 and over reached one billion in 2020 and is projected to grow to 1.4 billion by 2030 (World Health Organization, 2024). Aging is often accompanied by a decline in physical abilities and an increased susceptibility to various diseases. In the central nervous system (CNS), aging raises the risk of developing neurodegenerative diseases such as Alzheimer’s disease (AD) and Parkinson’s disease (PD) (Hou et al., 2019). A prevailing hypothesis suggests that aging is associated with disruptions in the homeostatic environment of the brain, leading to the deterioration of neuronal and synaptic functions (Allen et al., 2023). The maintenance of brain tissue homeostasis primarily depends on the interactions among neurons and the supporting glial cells including microglia, astrocytes, and oligodendrocytes (Procès et al., 2022). In recent decades, mounting evidence has revealed spatial and temporal genetic changes in these cells during aging, aiming to identify their potential association with age-related neurodegeneration (Allen et al., 2023; Consortium, 2020).

Microglia, the primary immune cells of CNS, play a critical role in maintaining brain tissue homeostasis (Li et al., 2023). They are responsible for eliminating harmful substances, damaged cells, and redundant synapses. Additionally, they release pro-inflammatory cytokines to aid in defense against infections and in response to injury (Colonna and Butovsky, 2017). However, with aging, accumulated oxidative stress and mitochondrial dysfunction impair microglial function, leading to either a dystrophic/senescent state or an over-reactive phenotype, as observed in aged human and animal brains (Malvaso et al., 2023). In aged individuals, microglia exhibit altered morphology, characterized by shortened processes and reduced branching in the gray matter of humans and the white matter of rodents (Antignano et al., 2023; Davies et al., 2017). These aged microglia adopted a primed phenotype, marked by decreased expression of homeostatic genes such as CD200, CX3CL1, and CD47, alongside increased expression of stimulatory genes, including MHC, CD86, CD68, CR3, and TLRs (Antignano et al., 2023). Furthermore, aged microglia showed a reduced ability to clear neuronal debris, apoptotic bodies, and secreted proteins (Gabandé-Rodríguez et al., 2020). Age-related microglial dysfunction is likely driven by shifts in key regulatory genes, such as CEBPβ and MEF2C, which modulate microglial homeostasis and reactivity. (Li et al., 2023).

CX3CR1 is a chemokine receptor predominantly expressed in microglia within the CNS, while its sole ligand, CX3CL1, is constitutively produced by neurons (Subbarayan et al., 2022). The full-length isoform of CX3CL1 mediates microglial cell adhesion, whereas its soluble isoform functions as a chemokine (Pawelec et al., 2020). Under physiological conditions, CX3CL1/CX3CR1 signaling is crucial for synaptic pruning by microglia during postnatal brain maturation: CX3CR1-deficient neonatal mice exhibit an excess of synapses compared to wild type mice (Mecca et al., 2018). Furthermore, CX3CR1 may play a role in maintaining microglial quiescence, as CX3CL1 attenuates microglial activation (Finneran et al., 2023). Recent years have seen an increase in studies exploring the roles of CX3CL1/CX3CR1 signaling in aging-related neurodegenerative diseases, including AD and PD (Pawelec et al., 2020). Emerging evidence indicates that disrupted CX3CL1/CX3CR1 signaling can exert either beneficial or detrimental effects on disease progression. These opposing outcomes likely stem from differences in pathological contexts across disease types or experimental models (Pawelec et al., 2020). Given that aging is a common risk factor for neurodegenerative diseases, investigating the roles of CX3CL1/CX3CR1 signaling during natural aging could enhance our understanding of their implications in aging-related conditions. Previous reports have indicated dysregulation of the CX3CL1/CX3CR1 axis in aging, evidenced by decreased CX3CL1 level in the aged rat brain and downregulated CX3CR1 expression in the microglia of aged mouse brain (Bachstetter et al., 2011; Fritze et al., 2024). This phenomenon may be linked to microglial activation and cognitive decline associated with aging (Bachstetter et al., 2011; Li et al., 2023). However, current aging studies primarily focus on the late age stage (18–24 months old), leaving the roles of CX3CL1/CX3CR1 axis during early aging (6–12 months old) and their underlying implications largely unexplored.

In this study, we assessed the expression level of the CX3CL1/CX3CR1 axis in the brains of middle-aged (10 months old) mice and explored their roles in early aging by knocking out CX3CR1. We evaluated microglial activation and conducted proteomic analysis to identify the effects of CX3CR1 knockout. To validate our evaluation methods, we included 5xFAD mice as a positive control group for microglial activation and neuronal impairment.

## 2 Materials and methods

### 2.1 Transgenic mice

Homozygous CX3CR1^GFP/GFP^ mice (stock No. 005582) were purchased from the Jackson Laboratory (Bar Harbor, ME, United States). With an enhanced green fluorescent protein sequence inserted in the coding exon of CX3CR1 gene, CX3CR1^GFP/GFP^ mice express no wild type CX3CR1 mRNA (Jung et al., 2000). 5xFAD mice (MMRRC stock No. 34848) were purchased from the Jackson Laboratory and maintained on a C57BL/6J genetic background by backcrossing with C57BL/6J wild type mice (Jawhar et al., 2012). C57BL/6J mice were obtained from the Laboratory Animal Unit of the University of Hong Kong and used as control animals. Only female mice were used in this study. All animals were housed in a temperature controlled room with a 12-h light/dark cycle. All animals were handled according to the ARRIVE guidelines. The animal groups and the number of mice used in each group were as follows: 3 months old C57BL/6J mice (young WT, n = 10), 10 months old C57BL/6J mice (middle age WT, n = 10), 10 months old CX3CR1^GFP/GFP^ mice (middle age KO, n = 10), and 10 months old 5xFAD mice (AD, n = 10).

### 2.2 Quantitative RT-PCR

Total RNA was extracted from the hippocampus and frontal cortex using Trizol (Thermo Fisher Scientific, Waltham, MA, United States) following manufacturer’s instructions. RNA concentration was measured using a NanoDrop 2000 (Thermo Fisher Scientific). RNA (1 µg) was reverse transcribed into cDNA in a total reaction volume of 20 µL using PrimeScript RT Master Mix (Takara, Kusatsu, Shiga, Japan). Quantitative PCR was performed on the cDNAs with a SYBR Green PCR reagent (Roche, San Francisco, CA, United States) on a CFX Connect Real-Time PCR System (Bio-Rad, Hercules, CA, United States). The primers for the detected genes are listed in Table 1. The relative mRNA expression levels of the target genes were calculated using the 2^−ΔΔCT^ method with β-actin as the reference gene. Six animals from each experimental group were analyzed for this task.

**TABLE 1:** Primer sequences used for qRT-PCR.

Gene	Forward primer (5′-3′)	Reverse primer (5′-3′)
β-actin	GTG​ACG​TTG​ACA​TCC​GTA​AAG​A	GCC​GGA​CTC​ATC​GTA​CTC​C
TNF-α	CCT​GTA​GCC​CAC​GTC​GTA​G	GGG​AGT​AGA​CAA​GGT​ACA​ACC​C
IL-1α	CGA​AGA​CTA​CAG​TTC​TGC​CAT​T	GAC​GTT​TCA​GAG​GTT​CTC​AGA​G
IL-1β	CTG​TGA​CTC​ATG​GGA​TGA​TGA​TG	CGG​AGC​CTG​TAG​TGC​AGT​TG
CX3CR1	GAG​TAT​GAC​GAT​TCT​GCT​GAG​G	CAG​ACC​GAA​CGT​GAA​GAC​GAG
CX3CL1	ACG​AAA​TGC​GAA​ATC​ATG​TGC	CTG​TGT​CGT​CTC​CAG​GAC​AA
CD68	CCA​TCC​TTC​ACG​ATG​ACA​CCT	GGC​AGG​GTT​ATG​AGT​GAC​AGT​T
MARCO	CTG​TGG​CAA​TGG​ATC​ACT​AGC	CTC​CTG​GCT​GGT​ATG​GAC​C
CD36	GAA​CCA​CTG​CTT​TCA​AAA​ACT​GG	TGC​TGT​TCT​TTG​CCA​CGT​CA
SRA	TGA​ACG​AGA​GGA​TGC​TGA​CTG	GGA​GGG​GCC​ATT​TTT​AGT​GC
SRB1	TTT​GGA​GTG​GTA​GTA​AAA​AGG​GC	TGA​CAT​CAG​GGA​CTC​AGA​GTA​G
CD36	GAA​CCA​CTG​CTT​TCA​AAA​ACT​GG	TGC​TGT​TCT​TTG​CCA​CGT​CA
RAGE	ACT​ACC​GAG​TCC​GAG​TCT​ACC	GTA​GCT​TCC​CTC​AGA​CAC​ACA
SYP	CAG​TTC​CGG​GTG​GTC​AAG​G	ACT​CTC​CGT​CTT​GTT​GGC​AC
PSD95	TGA​GAT​CAG​TCA​TAG​CAG​CTA​CT	CTT​CCT​CCC​CTA​GCA​GGT​CC

### 2.3 Immunofluorescence staining

Mouse brain tissues were fixed in 4% paraformaldehyde for 48 h, dehydrated, and embedded in paraffin. The tissue blocks were then sectioned into 5 µm thick slices (sagittal sections). For immunofluorescence staining, the sections were subjected to dewaxing, rehydration, and antigen retrieval. After rinsing in PBS for three times, the sections were blocked by immersing in 10% goat serum in PBS supplemented with 0.5% triton X-100 for 1 h at room temperature. Afterward, the sections were incubated with primary antibodies including rabbit anti-Iba-1 (1:500, WAKO, Chou-ku, Osaka, Japan), rabbit anti-GFAP (1:500, Abcam, Cambridge, UK), and rabbit anti-synaptophysin (1:500, Abcam) overnight at 4°C. After rinsing in PBS for three times, the sections were incubated with Alexa-568 fluorescent-conjugated goat anti-rabbit IgG secondary antibody (1:500, Thermo Fisher Scientific) for 1 h at room temperature. Finally, the sections were counterstained with DAPI (1:1000) for 5 min. Images were captured with an ECLIPSE Ti2-E confocal microscope (Nikon, Nishioi, Shinagawa-ku, Tokyo). Fluorescence intensity was calculated by ImageJ software (National Institutes of Health, Bethesda, Maryland, United States). Four animals from each experimental group were analyzed for this task.

### 2.4 Morphological analysis of microglia and astrocyte

High-quality images of Iba-1 stained microglia and GFAP stained astrocytes were captured using ×63 objective in a confocal microscope. Z-stack images were obtained from fixed positions in the CA1, CA3, and DG regions of the hippocampus, as well as the frontal cortex, and subsequently converted to 2D images via maximum intensity projection in NIS-Elements (Nikon). Iba-1 and GFAP positive cells with DAPI-stained nuclei were counted using ImageJ software, selecting cells with relatively intact morphology for further morphological analysis. For each selected cell, the size of cell body and length of each cell process were measured, and the number of processes was counted manually in ImageJ. The following microglia cell counts and animal numbers were analyzed: hippocampus (merged from CA1, CA3, and DG): young WT (66 cells from 4 mice), middle age WT (49 cells from 3 mice), middle age KO (45 cells from 4 mice), 5xFAD (86 cells from 3 mice); frontal cortex: young WT (46 cells from 4 mice), middle age WT (23 cells from 3 mice), middle age KO (35 cells from 4 mice), 5xFAD (39 cells from 3 mice). The following astrocyte cell counts were analyzed: hippocampus (merged from CA1, CA3, and DG): young WT (42 cells from 3 mice), middle age WT (37 cells from 3 mice), middle age KO (35 cells from 3 mice), 5xFAD (39 cells from 3 mice).

### 2.5 Protein extraction from formalin-fixed paraffin-embedded cerebral tissue

Total proteins were extracted from the formalin-fixed paraffin-embedded cerebral tissues following a previously published protocol (Weke et al., 2022). Briefly, 10 µm brain sections mounted on slides (5 sections were included for each mouse) were deparaffinized by two xylene washes and subsequently rehydrated by serial ethanol and distilled water treatment. The cerebral tissue from the brain sections was then transferred into a clean collection tube by scratching them off from the glass slides. The samples were added with extraction buffer (100 μL buffer consisting of 50 μL of 30% Acetonitrile, 100 mM Ammonium Bicarbonate buffer, 45 μL of 8 M urea, and 5 μL of 1% RapiGest) and incubated at 95°C for 90 min. Afterwards, the samples were added with 700 mM dithiothreitol and incubated at 37°C for 30 min to reduce disulfide bonds, and followingly added with 700 mM iodoacetamide and incubated at 37°C for 30 min to allow alkylation. The purity and integrity of protein samples were determined by SDS-PAGE electrophoresis. Protein quantification was carried out using a BCA kit (Thermo Fisher Scientific) following the manufacturer’s instructions. Three animals from each experimental group were analyzed for this task. The measured protein concentrations were as follows: young WT (1.763, 1.695, and 1.924 μg/μL), middle age WT (1.731, 2.166, and 2.098 μg/μL), middle age KO (2.148, 2.381, 1.992 μg/μL), and AD (1.800, 1.947, 2.070 μg/μL).

### 2.6 Liquid chromatography-mass spectrometry (LC-MS) analysis

Protein samples were added with sequencing-grade trypsin (1 μg/μL, sigma) and incubated at 37°C for 12 h for digestion. The resulting peptides were desalted on SOLA™ SPE 96-plate Column (Thermo Fisher Scientific). Peptides were desalted with 5% methanol and eluted with 100% methanol. After vacuum dried, the samples were resuspended with iRT peptides (1:10). LC-MS was conducted by a timsTOF^TM^ Pro mass spectrometer (Bruker, Billerica, MA, United States) equipped with an EASY-Spray™ Source (Thermo Fisher Scientific). Samples were loaded and separated by a C18 column on an EASY-nLC^TM^ 1200 system (Thermo Fisher Scientific) at a flow rate of 300 nL/min. The linear gradient program was as follows: 0–20 min, 5%–22% B; 20–24 min, 22%–37% B; 24–27 min, 37%–80% B; 27–30 min, 80% B. The ion mobility was set between 0.7 and 1.3 Vs/cm^2^, with collision energy ranging from 20 to 59 eV. The MS spectra were recorded at a scan range of 100–1700 m/z.

### 2.7 MS data analysis

MS spectra were processed with Spectronaut Pulsar™ 18.4 (Biognosys, Schlieren, Switzerland). A reviewed Uniprot Mus Musculus proteome database (downloaded on February 1, 2024) was used. Carbamidomethylation (C) was set as a fixed modification, while oxidation (M) and acetylation (protein N-term) were set as variable modifications. The Precursor and Protein Q value cutoffs were set at 0.01, and Quantity MS-Level was set at MS2. In total, 5,692 proteins were identified from mouse cerebral tissues. Differentially expressed proteins (DE-proteins) were identified based on a log_2_(fold change) threshold of >0.3 or < -0.3 and a P-value of <0.05. All identified proteins were annotated using Gene Ontology (GO) (http://www.blast2go.com/b2ghome; http://geneontology.org/) and KEGG pathway databases (http://www.genome.jp/kegg/). Enrichment analysis for GO and KEGG pathways was conducted on the DE-proteins. Protein-protein interaction analysis was performed using STRING (https://string-db.org/).

### 2.8 Statistical analysis

Statistical analysis was performed using IBM SPSS Statistics 20 (Chicago, IL, United States). For comparisons between two groups, a two-tailed Student’s t-test was employed. When comparing multiple groups, one-way ANOVA was utilized, followed by Fisher’s Least Significant Difference test for *post hoc* comparisons, or Dunnett’s test in cases where group variances were unequal. Graphical representations of the data were generated with GraphPad Prism 8.0 (San Diego, CA, United States). Results are expressed as mean ± SD. Statistical significance was determined at *p < 0.05.

## 3 Results

### 3.1 Elevated CX3CR1 expression in the middle-aged mouse brain

**FIGURE 1:**
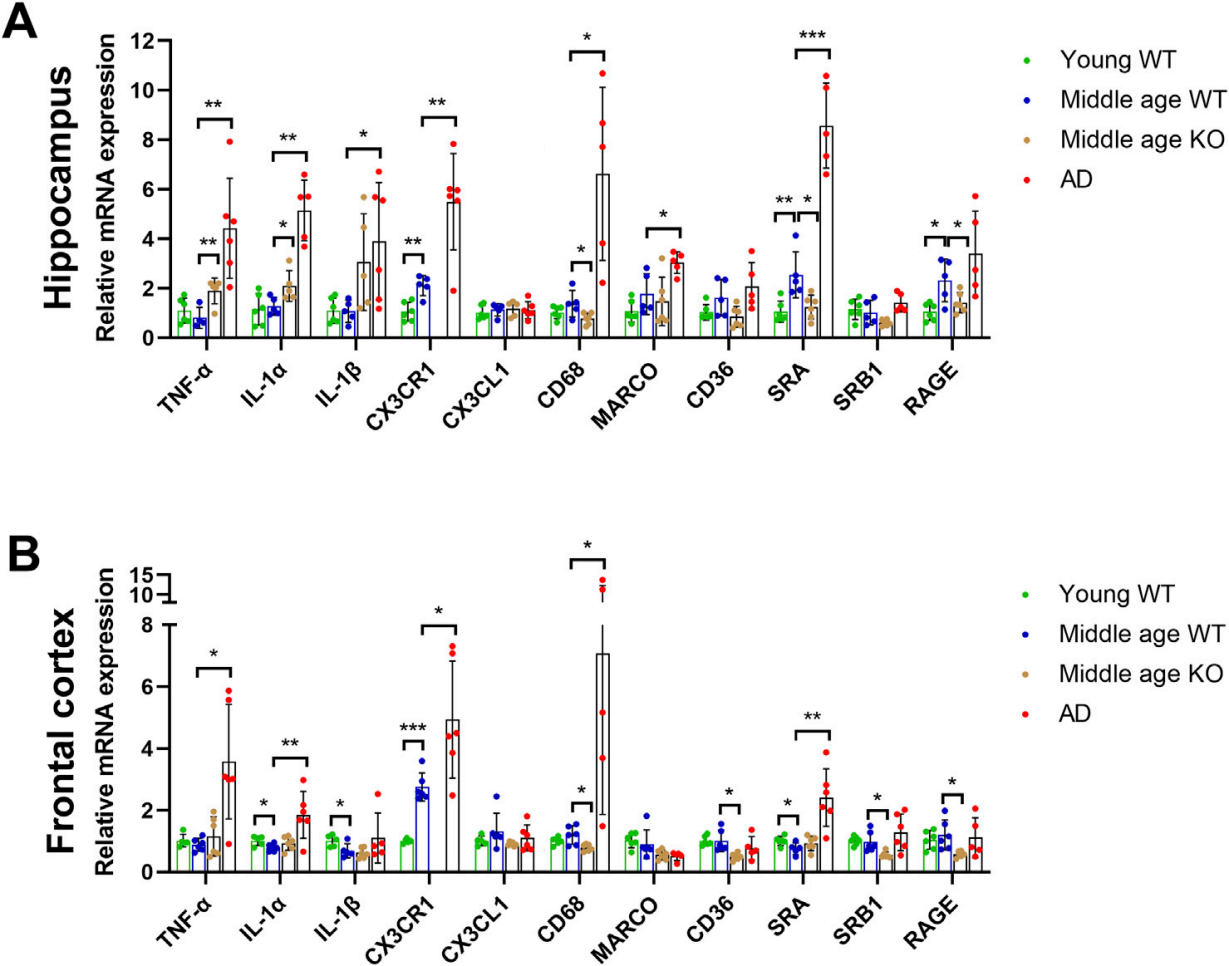
CX3CR1 deficiency upregulated pro-inflammatory cytokines and downregulated scavenger receptors in the middle-aged mouse brain. **(A)** Cytokine and scavenger receptor mRNA levels in the hippocampus of young adult (young WT), middle-aged C57BL/6J mice (middle age WT), CX3CR1-deficient mice (middle age KO), and 5xFAD mice (AD). CX3CR1, SRA, and RAGE were upregulated in the hippocampus of middle-aged mice, whereas CX3CR1 deficiency upregulated TNF-α and IL-1α while downregulating CD68, SRA, and RAGE. In contrast, TNF-α, IL-1α, IL-1β, CX3CR1, CD68, MARCO, and SRA were upregulated in the 5xFAD mice. **(B)** Cytokine and scavenger receptor mRNA levels in the frontal cortex of the indicated groups. CX3CR1 was upregulated, while IL-1α, IL-1β, and SRA were downregulated in the frontal cortex of middle-aged mice, whereas CX3CR1 deficiency downregulated CD68, CD36, SRB1, and RAGE. In contrast, TNF-α, IL-1α, CX3CR1, CD68, and SRA were upregulated in the 5xFAD mice. *P < 0.05; **P < 0.01; ***P < 0.001.

To investigate the roles of CX3CL1/CX3CR1 signaling pathway in the early stage of aging, its gene expression was evaluated in the hippocampus and frontal cortex of middle-aged mice (10 months old), using young adult mice (3 months old) as controls. Notably, CX3CR1 significantly increased by 2.11 ± 0.41-fold (**P = 0.002) in the hippocampus (Figure 1A) and by 2.75 ± 0.46-fold (***P < 0.001) in the frontal cortex (Figure 1B) of middle-aged mice compared to young controls, though CX3CL1 remained unchanged in both tissues. Given the crucial role of CX3CR1 in regulating activation and phagocytosis of microglia (Subbarayan et al., 2022), we continued to detect the pro-inflammatory cytokines, including TNF-α, IL-1α, and IL-1β, as well as the scavenger receptors, including CD68, macrophage receptor with collagenous structure (MARCO), CD36, scavenger receptor A (SRA), scavenger receptor class B type 1 (SRB1), and receptor for advanced glycation end-products (RAGE) in the hippocampus and frontal cortex. As a result, TNF-α, IL-1α, and IL-1β were not altered in the hippocampus (Figure 1A), whereas IL-1α and IL-1β were significantly decreased to 0.79 ± 0.12 (*P = 0.024) and 0.69 ± 0.23-fold (*P = 0.041), respectively, in the frontal cortex of middle-aged mice compared to young mice (Figure 1B). Furthermore, we found that SRA and RAGE expression levels were significantly increased by 2.55 ± 0.93 (**P = 0.006) and 2.32 ± 0.86 -fold (*P = 0.026), respectively, in the hippocampus of middle-aged mice than the young controls (Figure 1A). This may suggest enhanced microglial phagocytic activity, as SRA and RAGE play key roles in microglial phagocytosis and activation by binding to ligands such as β-amyloid (Aβ) and advanced glycation end products, thereby contributing to the pathogenesis of brain diseases like AD (Wilkinson and El Khoury, 2012). Unlike in the hippocampus, the SRA level in the frontal cortex of middle-aged mice was significantly decreased to 0.76 ± 0.19-fold (*P = 0.033) than its level in young mice (Figure 1B).

### 3.2 CX3CR1 deficiency upregulated pro-inflammatory cytokines and downregulated scavenger receptors in the middle-aged mouse brain

To determine whether the upregulated CX3CR1 affected the expressions of pro-inflammatory cytokines and scavenger receptors in the brains of middle-aged mice, we knocked out CX3CR1 and subsequently performed qRT-PCR analysis on the hippocampus and frontal cortex. Given the significant activation of microglia, the primary defender and scavenger of CNS, in AD mice, we used the brains of age-matched 5xFAD mice as a positive control. CX3CR1 was undetectable in both brain regions of the CX3CR1 knockout mice but was increased by 2.62 ± 0.93-fold (**P = 0.004) in the hippocampus and 1.81 ± 0.70-fold (*P = 0.036) in the frontal cortex of 5xFAD mice compared to age-matched wild type mice. CX3CL1 level, on the other hand, remained unchanged in both the CX3CR1 knockout mice and the 5xFAD mice (Figures 1A,B).

CX3CR1 knockout significantly upregulated TNF-α and IL-1α levels by 2.39 ± 0.65 (**P = 0.002) and 1.73 ± 0.51-fold (*P = 0.042), respectively, while not changing IL-1β level in the hippocampus of middle-aged mice (Figure 1A). Whereas, CX3CR1 knockout did not alter the expressions of TNF-α, IL-1α, and IL-1β in the frontal cortex (Figure 1B). In the AD control mice, significantly increased TNF-α (**P = 0.003), IL-1α (**P = 0.001), and IL-1β (*P = 0.032) were detected in the hippocampus while significantly increased TNF-α (*P = 0.016) and IL-1α (**P = 0.007) were observed in the frontal cortex (Figures 1A,B).

In contrast to the increase in cytokines, scavenger receptors were downregulated by CX3CR1 knockout in the brains of middle-aged mice. In the hippocampus of CX3CR1 knockout mice, CD68, SRA, and RAGE were significantly decreased to 0.60 ± 0.18 (*P = 0.033), 0.51 ± 0.20 (*P = 0.015), and 0.65 ± 0.19-fold (*P = 0.047), respectively, compared to the wild type controls (Figure 1A). Similarly, in the frontal cortex of CX3CR1 knockout mice, CD68, CD36, SRB1, and RAGE were significantly decreased to 0.68 ± 0.10 (*P = 0.011), 0.51 ± 0.11 (*P = 0.014), 0.59 ± 0.11 (*P = 0.023), and 0.52 ± 0.09-fold (*P = 0.025), respectively, compared to the wild type controls (Figure 1B). In the AD control mice, scavenger receptors were upregulated in response to Aβ deposition, as indicated by increased levels of CD68 (*P = 0.028), MARCO (*P = 0.023), and SRA (***P < 0.001) in the hippocampus, as well as increased levels of CD68 (*P = 0.021) and SRA (**P = 0.004) in the frontal cortex (Figures 1A,B).

### 3.3 CX3CR1 deficiency altered microglial morphology in the middle-aged mouse brain

**FIGURE 2:**
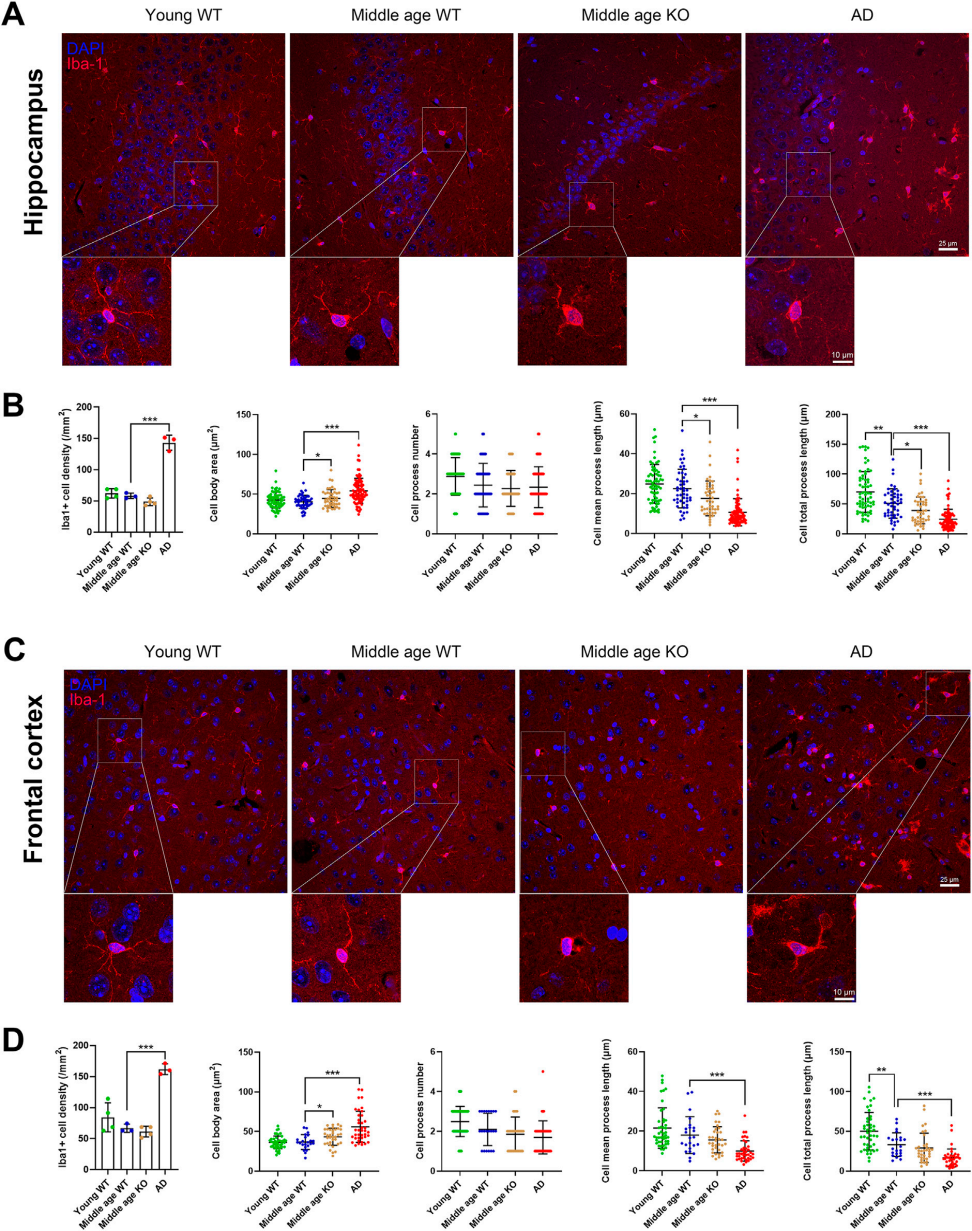
CX3CR1 deficiency altered microglial morphology in the middle-aged mouse brain. **(A)** Representative Iba-1 positive microglial images in the hippocampus from young adult, middle-aged, CX3CR1-deficient, and 5xFAD mice. **(B)** CX3CR1 deficiency increased microglial cell body area while decreasing mean process length and total process length in the hippocampus of middle-aged mice, without affecting cell number. In contrast, 5xFAD mice showed increased microglial cell number, enlarged cell body area, and shortened processes. **(C)** Representative Iba-1 images from the frontal cortex of the indicated groups. **(D)** CX3CR1 deficiency increased microglial cell body area in the frontal cortex of middle-aged mice. In contrast, 5xFAD mice showed increased microglial cell number, enlarged cell body area, and shortened processes. *P < 0.05; **P < 0.01; ***P < 0.001.

The observed changes in pro-inflammatory cytokines and scavenger receptors in the brains of CX3CR1 knockout mice suggest a potential alteration in microglial state. To investigate this, we quantified the number of Iba-1-positive microglia and analyzed their morphological parameters, including cell body area, number of processes, mean process length, and total process length. Compared with the young mice, the middle-aged mice showed no significant change in microglial cell density, cell body area, and mean process length in both the hippocampus and frontal cortex. However, the total process length of microglia significantly decreased in the hippocampus from 70.07 ± 34.19 (young mice) to 50.71 ± 24.62 µm (**P = 0.001, Figures 2A,B), and in the frontal cortex from 50.07 ± 23.67 (young mice) to 33.17 ± 14.84 µm (**P = 0.003, Figures 2C,D) in the middle-aged mice. When CX3CR1 was knocked out in the middle-aged mice, microglial cell density was unaltered in both the hippocampus and frontal cortex. However, microglial cell body area was significantly increased in the hippocampus from 40.35 ± 8.19 (middle-aged mice) to 44.54 ± 11.06 µm^2^ (*P = 0.039, Figures 2A,B) and in the frontal cortex from 36.71 ± 9.30 (middle-aged mice) to 43.32 ± 10.75 µm^2^ (*P = 0.019, Figures 2C,D). Additionally, cell mean process length and total process length were significantly decreased from 22.55 ± 9.69 (middle-aged mice) to 17.65 ± 8.71 µm (*P = 0.012) and from 50.71 ± 24.62 (middle-aged mice) to 39.01 ± 22.49 µm (*P = 0.020), respectively, in the hippocampus (Figures 2A,B). To conclude, CX3CR1 deficiency led to a microglial morphology characterized by an enlarged cell body area and shortened processes, likely indicating an activated state in the brain. As a control, AD mice presented similar changes in microglial morphology, including an increased cell body area and reduced mean and total process lengths in the hippocampus and frontal cortex (Figures 2A–D).

### 3.4 CX3CR1 deficiency altered astrocyte morphology in the hippocampus of middle-aged mouse

**FIGURE 3:**
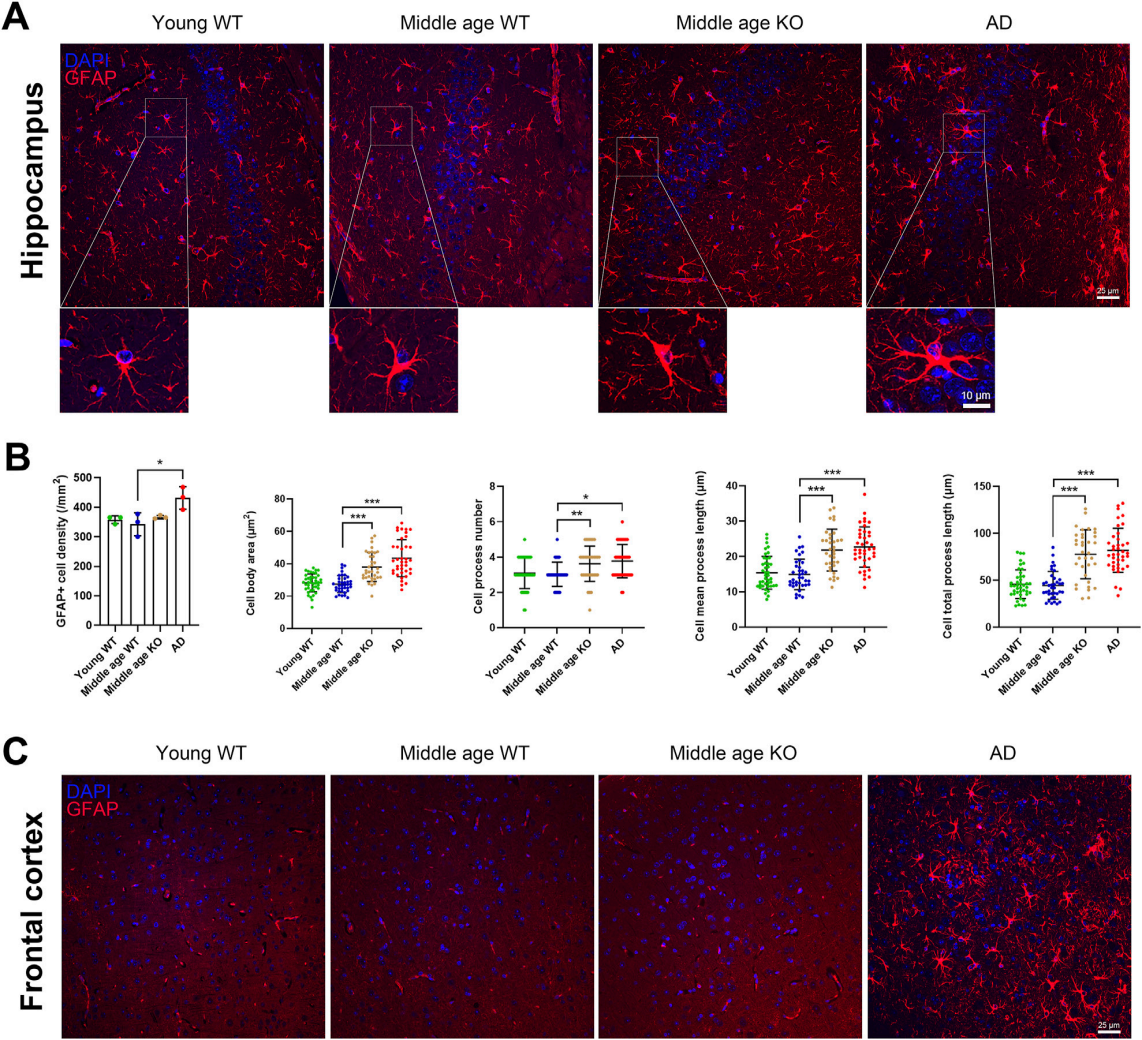
CX3CR1 deficiency altered astrocyte morphology in the hippocampus of middle-aged mouse. **(A)** Representative GFAP positive astrocyte images in the hippocampus from young adult, middle-aged, CX3CR1-deficient, and 5xFAD mice. **(B)** CX3CR1 deficiency increased astrocyte cell body area, process number, mean process length, and total process length in the hippocampus of middle-aged mice, without affecting cell number. In contrast, 5xFAD mice showed increased cell number along with similar morphological changes. **(C)** Representative GFAP images from the frontal cortex. Few GFAP positive cells were observed in young adult, middle-aged, and CX3CR1-deficient mice. *P < 0.05; **P < 0.01; ***P < 0.001.

Astrocytes extensively interact with other CNS cell types, including neurons and microglia, and play crucial roles in ion homeostasis and synaptic modulation (Baldwin et al., 2024). In response to injury or pathological processes, astrocytes become reactive and hypertrophic, leading to an increase in cell number and the length of GFAP-positive processes (Zhou et al., 2019). Here, we investigated whether CX3CR1 knockout influences astrocyte reactivity in the brains of middle-aged mice. We quantified the number of GFAP-positive astrocytes and analyzed their morphological parameters, including cell body area, number of processes, mean process length, and total process length. No significant changes were observed in astrocyte density or morphology in the hippocampus of middle-aged mice compared to young mice (Figures 3A,B). When CX3CR1 was knocked out in the middle-aged mice, astrocyte density in the hippocampus remained unchanged, while the cell body area, number of processes, mean process length, and total process length were significantly increased from 27.49 ± 5.26 to 38.05 ± 9.01 µm^2^ (***P < 0.001), from 3.03 ± 0.69 to 3.63 ± 1.00 (**P = 0.008), from 14.92 ± 4.30 to 21.82 ± 5.92 µm (***P < 0.001), and from 44.54 ± 14.77 to 77.68 ± 26.01 µm (***P < 0.001), respectively (Figures 3A,B). In contrast, AD mice showed an increased number of astrocytes, along with increased cell body area, mean process length, and total process length in the hippocampus (Figures 3A,B). In the frontal cortex, GFAP-positive astrocytes were scarcely observed in the wild-type and CX3CR1 knockout mice (Figure 3C). In contrast, a massive presence of astrocytes was observed in the frontal cortex of AD mice, ruling out the possibility of technical issues. Previous studies have reported that GFAP immunostaining estimates only ∼15% of an astrocyte’s surface area, which may explain the unexpectedly weak GFAP signal (Baldwin et al., 2024).

### 3.5 Middle-aged mouse cerebrum showed altered synaptic and translational protein profiles

**FIGURE 4:**
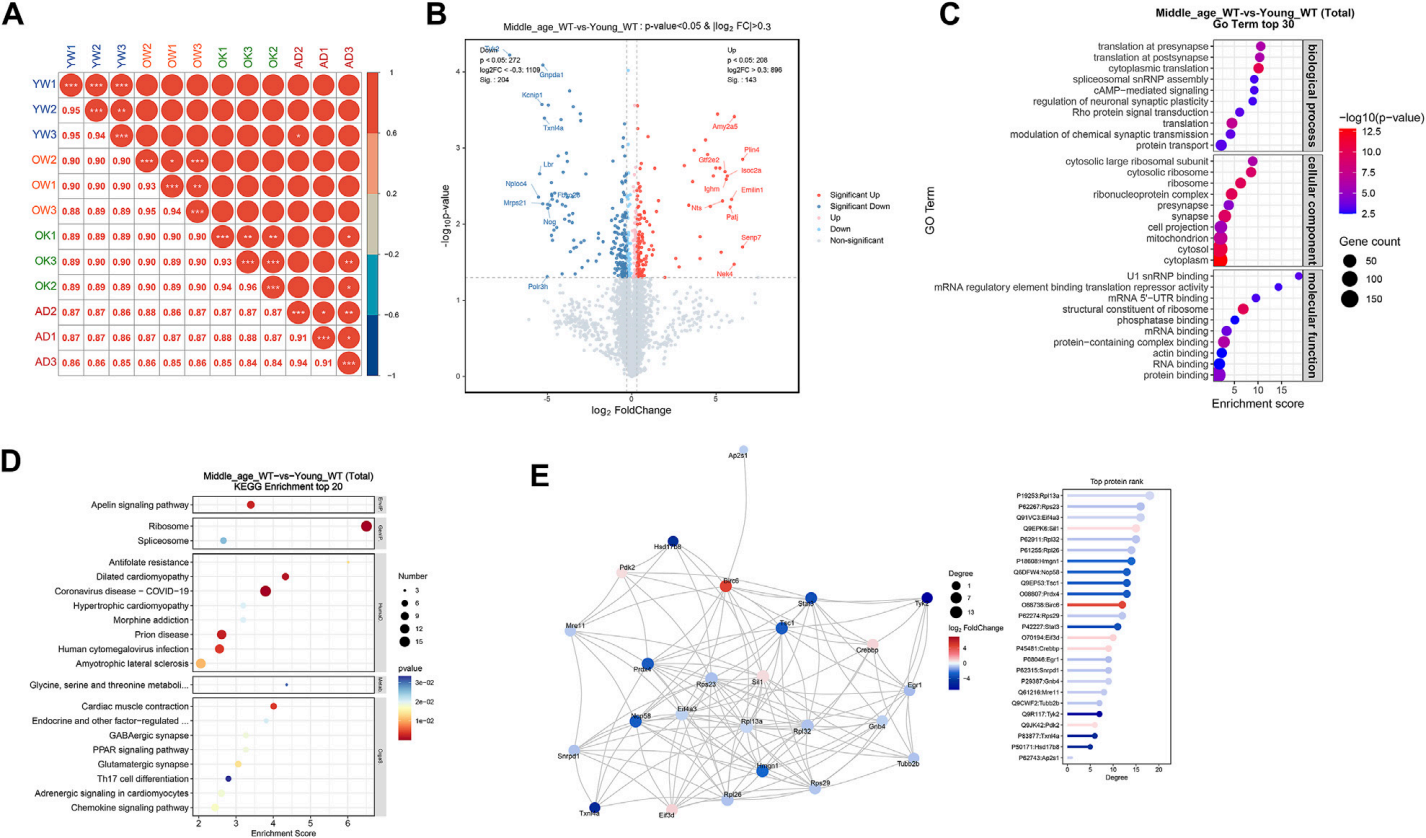
Middle-aged mouse cerebrum showed altered synaptic and translational protein profiles. **(A)** Proteomic analysis of proteins extracted from the cerebrum of young adult (YW), middle-aged (OW), CX3CR1-deficient (OK), and 5xFAD (AD) mice. Sample correlation analysis showed high intra-group correlations. **(B)** Volcano map illustrating DE-proteins in middle-aged mice compared to young controls. **(C)** GO enrichment analysis of DE-proteins, displaying the top 30 pathways. **(D)** KEGG enrichment analysis of DE-proteins, displaying the top 20 pathways. **(E)** PPI network analysis of DE-proteins.

To unveil the changes in protein expression in the brains of middle-aged mice, proteomic profiling was conducted on the cerebrum of 10 months old mice, with 3 months old mice as young controls. The sample corrplot illustrated high intragroup correlations, indicating consistent protein profiles within each group (Figure 4A). Differential expression analysis identified 143 upregulated proteins (log_2_FC > 0.3, P < 0.05) and 204 downregulated proteins (log_2_FC < −0.3, P < 0.05) in the cerebrum of middle-aged mice compared to young controls (Figure 4B; Supplementary Table S1). Subsequent GO enrichment analysis of the DE-proteins identified synaptic homeostasis related pathways, including “regulation of neuronal synaptic plasticity” and “synapse”, as well as translation related pathways, including “translation” and “ribosome” (Figure 4C; Supplementary Table S2). KEGG analysis further confirmed the enrichment of DE-proteins in “GABAergic synapse”, “glutamatergic synapse”, and “ribosome” pathways (Figure 4D; Supplementary Table S3). To gain a deeper understanding of the biological functions of these DE-proteins, we conducted a protein-protein interaction (PPI) network analysis. The top ranked proteins, identified based on their high degree centrality in the PPI network, included the ribosome proteins such as Rpl13a, Rps23, Rpl32, Rpl26, and Rps29, as well as the translation initiation factor Eif3d (Figure 4E). These proteins were associated with GO pathways including “synapse” and “translation” (Supplementary Table S2). To conclude, multiple proteins involved in synaptic homeostasis and translation were altered in the cerebrum of middle-aged mice.

### 3.6 CX3CR1 deficiency disrupted synaptic and RNA processing proteins in the middle-aged mouse cerebrum

**FIGURE 5:**
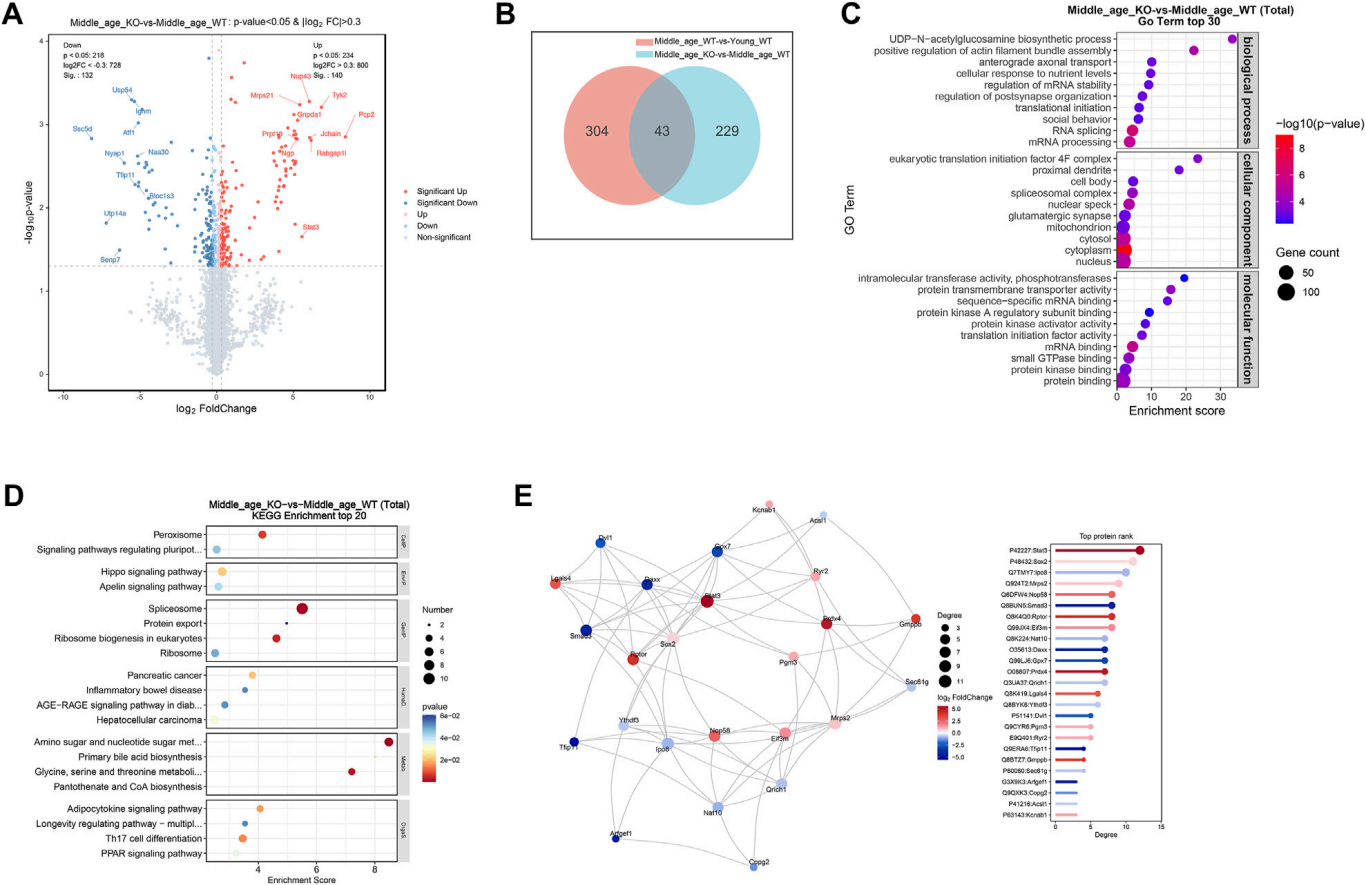
CX3CR1 deficiency altered synaptic and RNA processing protein profiles in the middle-aged mouse cerebrum. **(A)** Volcano map illustrating DE-proteins in the cerebrum of middle-aged CX3CR1-deficient mice than wild type controls. **(B)** Venn diagram demonstrating the number of DE-proteins shared by middle-aged and CX3CR1-deficient mice. **(C)** GO enrichment analysis of DE-proteins, displaying the top 30 pathways. **(D)** KEGG enrichment analysis of DE-proteins, displaying the top 20 pathways. **(E)** PPI network analysis of DE-proteins.

To investigate how CX3CR1 modulates protein expression in the brains of middle-aged mice, we conducted proteomic profiling on the cerebrum of CX3CR1-deficient mice. For comparison, we also analyzed protein changes in the cerebrum of age-matched 5xFAD mice. The sample corrplot revealed high intragroup correlations (Figure 4A). Differential expression analysis identified 140 upregulated proteins (log2FC > 0.3, P < 0.05) and 132 downregulated proteins (log2FC < −0.3, P < 0.05) in the cerebrum of CX3CR1 knockout mice compared to wild type controls (Figure 5A; Supplementary Table S4). Venn analysis indicated that 43 DE-proteins identified in the middle-aged mice were further altered by CX3CR1 deficiency (Figure 5B; Supplementary Table S5). GO enrichment analysis of the DE-proteins in the CX3CR1-deficient mice demonstrated significant enrichment in pathways related to synaptic homeostasis, such as “glutamatergic synapse” and “synapse”, as well as RNA processing pathways, including “mRNA processing”, “RNA splicing”, and “mRNA binding” (Figure 5C; Supplementary Table S6). KEGG analysis revealed enrichment of these DE-proteins in pathways like “Peroxisome”, “Spliceosome”, “Ribosome”, and “Amino sugar and nucleotide sugar metabolism” (Figure 5D; Supplementary Table S7). PPI network analysis identified several proteins with a high degree centrality in the brains of CX3CR1 knockout mice, including the upregulated proteins Stat3, Sox2, Mrps2, Nop58, Rptor, and Prdx4, as well as the downregulated proteins lpo8, Smad3, Nat10, Daxx, and Gpx7 (Figure 5E).

**FIGURE 6:**
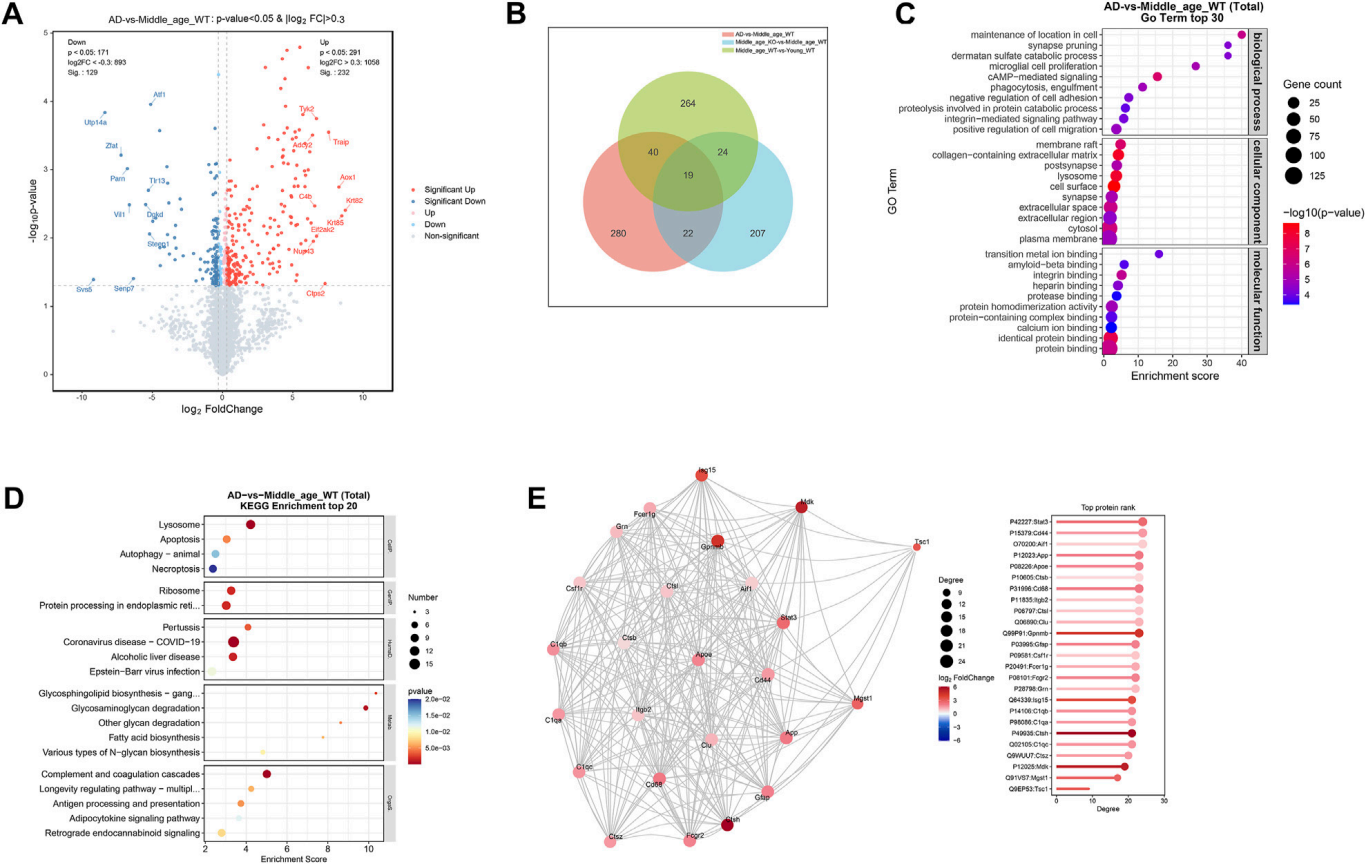
Protein profiles in the cerebrum of 5xFAD mice. **(A)** Volcano map illustrating DE-proteins in the cerebrum of 5xFAD mice. **(B)** Venn diagram demonstrating the number of DE-proteins shared among middle-aged, CX3CR1-deficient, and 5xFAD mice. **(C)** GO enrichment analysis of DE-proteins, displaying the top 30 pathways. **(D)** KEGG enrichment analysis of DE-proteins, displaying the top 20 pathways. **(E)** PPI network analysis of DE-proteins.

In the cerebrum of AD mice, a total of 232 proteins were found to be upregulated (log2FC > 0.3, P < 0.05), while 129 proteins were downregulated (log2FC < −0.3, P < 0.05) compared with the age matched wild type controls (Figure 6A; Supplementary Table S8). Venn analysis revealed that 41 DE-proteins were shared by the AD mice and CX3CR1-deficient mice (Figure 6B; Supplementary Table S9). Pathway enrichment analysis of the DE-proteins in AD mice indicated significant enrichment in GO pathways like “phagocytosis, engulfment”, “microglial cell proliferation”, “synapse”, “lysosome”, “amyloid-beta binding”, and “protein binding” (Figure 6C; Supplementary Table S10), and in KEGG pathways including “Lysosome”, “Apoptosis”, “Ribosome”, and “Complement and coagulation cascades” (Figure 6D; Supplementary Table S11). In the PPI network of AD mice, the top-ranked proteins were predominantly upregulated, including Stat3, Cd44, Aif1 (also known as Iba-1), App, Apoe, Cd68, Gfap, C1qb, and C1qa (Figure 6E).

### 3.7 CX3CR1 deficiency impaired synapse survival in the middle-aged mouse brain

**FIGURE 7:**
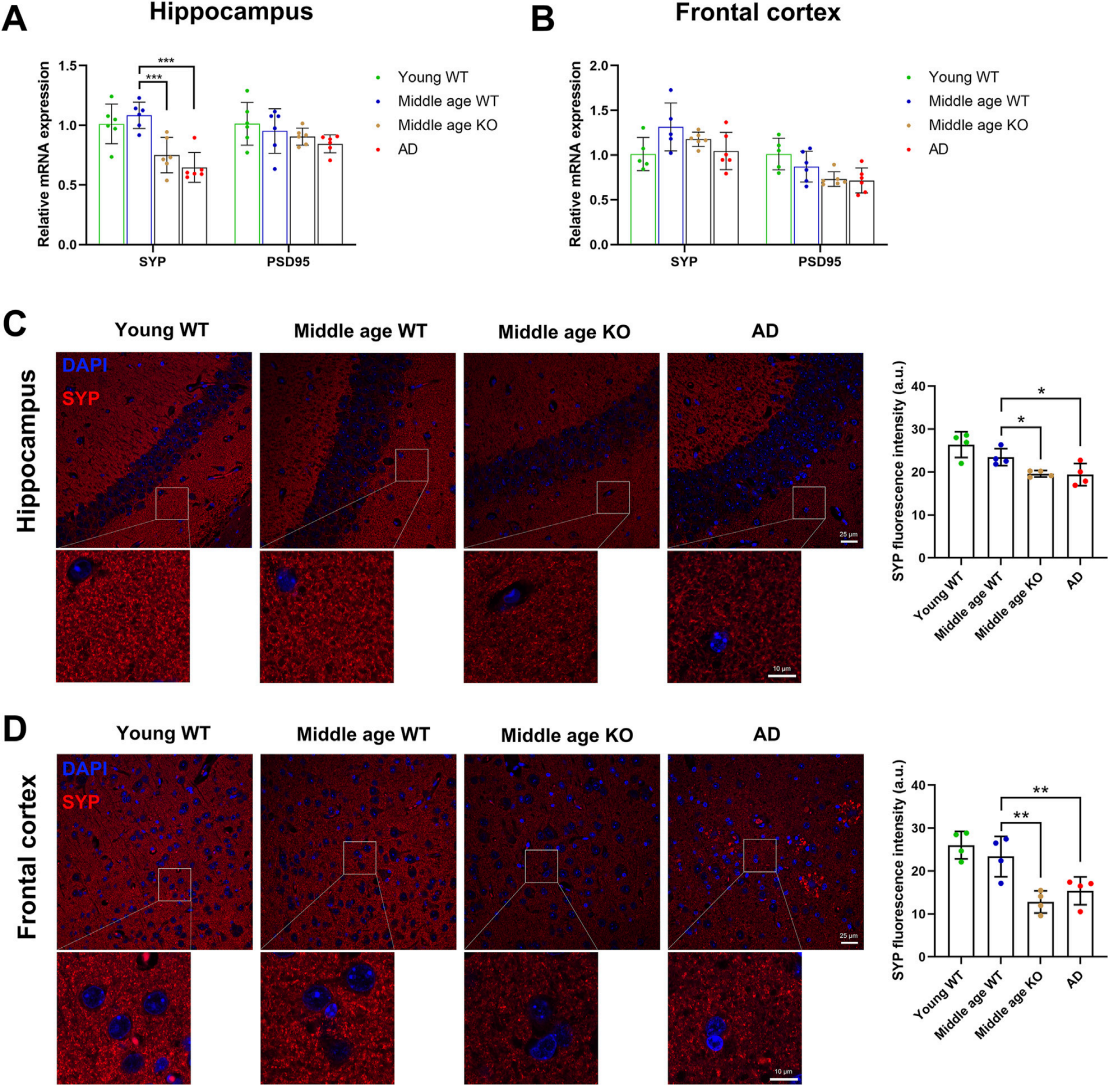
CX3CR1 deficiency impaired synaptic survival in the middle-aged mouse brain. **(A)** Synaptophysin (SYP) and PSD95 mRNA levels in the hippocampus of young adult, middle-aged, CX3CR1-deficient, and 5xFAD mice. Synaptophysin mRNA level was significantly lower in the hippocampus of CX3CR1-deficient and 5xFAD mice compared to wild type controls. **(B)** Synaptophysin and PSD95 mRNA levels in the frontal cortex of the indicated groups. **(C)** IHC staining showed decreased synaptophysin fluorescence intensity in the hippocampus of CX3CR1-deficient and 5xFAD mice versus controls. **(D)** IHC staining showed decreased synaptophysin fluorescence intensity in the frontal cortex of CX3CR1-deficient and 5xFAD mice versus controls. *P < 0.05; **P < 0.01; ***P < 0.001.

The identification of DE-proteins associated with synaptic homeostasis in the CX3CR1-deficient mice prompted us to ask whether CX3CR1 deficiency promotes or impairs the health of synapses. We evaluated the expression of the classical pre- and post-synaptic markers, synaptophysin and PSD95, respectively. Proteomic analysis revealed no significant alterations in cerebral levels of either marker, both with aging to middle age and in the context of CX3CR1 deficiency (Supplementary Tables S1 and S4). We next examined these markers in specific brain regions, the hippocampus and frontal cortex. Consistent with the proteomic findings, middle age did not significantly alter synaptophysin or PSD95 expression at either the transcriptional (Figures 7A,B) or translational (Figures 7C,D) levels in either brain region. Meanwhile, in the hippocampus, CX3CR1 deficiency significantly reduced the mRNA level of synaptophysin to 0.70 ± 0.14-fold (***P < 0.001) compared to wild type controls. This reduction was even more pronounced in AD mice, which showed a 0.60 ± 0.12-fold decrease (***P < 0.001; Figure 7A). In the frontal cortex, synaptophysin mRNA levels showed non-significant reductions in both CX3CR1-deficient mice (0.91 ± 0.06-fold) and AD mice (0.80 ± 0.16-fold) compared to wild-type controls (Figure 7B). PSD95 expression remained unaltered in both the hippocampus and frontal cortex of CX3CR1-deficient and AD mice compared to wild-type controls. (Figures 7A,B). Consistent with mRNA expression patterns, immunofluorescence analysis demonstrated significantly reduced synaptophysin-positive synapse density in both the hippocampus (*P = 0.032) and frontal cortex (**P = 0.001) of CX3CR1-deficient mice compared to wild-type controls. Similar synaptic deficits were observed in AD mice, with significant reductions in hippocampal (*P = 0.025) and frontal cortical (**P = 0.008) synapse density (Figures 7C,D).

## 4 Discussion

CX3CL1/CX3CR1 signaling has been well established as a contributor to aging-related neurodegenerative diseases (Subbarayan et al., 2022). Our study found that CX3CR1 was upregulated in the hippocampus and frontal cortex of middle-aged mice. These mice also exhibited increased levels of scavenger receptors, including SRA and RAGE, in the hippocampus, along with decreased levels of pro-inflammatory cytokines such as IL-1α and IL-1β in the frontal cortex. Additionally, we observed an enrichment of DE-proteins in pathways like “synapse,” “translation,” and “ribosome” in the cerebrum of middle-aged mice. Following CX3CR1 knockout, TNF-α and IL-1α levels were elevated, while CD68, SRA, and RAGE levels were decreased in the hippocampus. Microglial cells exhibited altered morphology, characterized by enlarged cell bodies and shortened processes, in the hippocampus and frontal cortex of CX3CR1-deficient mice, whereas astrocytes displayed morphological changes with enlarged cell bodies and elongated processes in the hippocampus. Moreover, DE-proteins induced by CX3CR1 knockout were enriched in pathways such as “glutamatergic synapse” and “RNA splicing”. Further investigation revealed a reduced density of synapses in both the hippocampus and frontal cortex of middle-aged CX3CR1-deficient mice.

Microglia are the resident macrophage-like immune cells of CNS. Recent evidence indicated microglia as a major contributor to brain aging (Hahn et al., 2023). Indeed, Aged microglia exhibit dystrophic morphology and increased expression of inflammatory markers, adopting a primed or senescent phenotype (Malvaso et al., 2023; Niraula et al., 2017). However, the molecular mechanisms regulating microglial susceptibility to aging remain unclear. The CX3CL1/CX3CR1 signaling pathway is essential for microglia-neuron communication. Early studies indicated that the CX3CL1/CX3CR1 axis in the brain could be altered with aging (Mecca et al., 2018). A recent study by Fritze et al. reported a decrease in CX3CR1 expression on microglia in the subventricular zone of aged mice (21–22 months old) compared with young controls (Fritze et al., 2024). In contrast, our study detected an upregulation of CX3CR1 gene expression in the hippocampus and frontal cortex of middle-aged mice (10 months old). Despite potential regional differences in microglial phenotypes, CX3CR1 likely plays varying roles throughout the lifespan of mice. CX3CL1/CX3CR1 signaling plays a key role in maintaining microglial quiescence (Mecca et al., 2018). Thus, the observed CX3CR1 upregulation may suppress microglial activity in middle-aged mice, as its knockout increased pro-inflammatory cytokine production and induced microglial retraction. Notably, microglia may upregulate the CX3CL1/CX3CR1 axis to autoregulate overactivation in pathological conditions like multiple sclerosis (Mecca et al., 2018).

As the primary defenders of the CNS, microglia continuously surveil their local environment, phagocytosing and clearing debris to maintain homeostasis (Petry et al., 2023). With aging, accumulated myelin debris may chronically challenge microglial phagocytic capacity (Rim et al., 2024). We observed an elevated expression of SRA and RAGE, two scavenger receptors expressed primarily in microglia, in the hippocampus of middle-aged mice. SRA promotes Aβ clearance, while RAGE mediates Aβ-induced microglial activation, both of which are implicated in AD pathogenesis (Wilkinson and El Khoury, 2012). Our findings suggest that microglia may enhance their phagocytic activity during middle age, though further validation is required. Notably, CX3CL1/CX3CR1 signaling plays critical roles in regulating microglial phagocytosis under pathological conditions, including AD and hypoxia (Puntambekar et al., 2022; Wang et al., 2023). Furthermore, CX3CR1 deficiency has been shown to impair the phagocytic activity of primary mouse microglia (Pawelec et al., 2020). In our study, CX3CR1 knockout led to decreased expression of scavenger receptors in the hippocampus and frontal cortex of middle-aged mice. These results strongly implicate the potential role of CX3CL1/CX3CR1 axis in preserving microglial phagocytic function during aging.

In the adult brain, the number of synapses typically remains constant, maintained by a delicate balance between formation and elimination, a process in which microglia play a pivotal role (Andoh and Koyama, 2021; Soteros and Sia, 2022). However, in the aging brain, synaptic structures begin to deteriorate (Radulescu et al., 2021). Notably, the presynaptic protein synaptophysin has been reported to be downregulated in the brains of middle-aged mice (10–12 months) when compared to young controls (Radulescu et al., 2021). In contrast, our study revealed no significant changes in the protein and mRNA levels of synaptophysin in either the hippocampus or the frontal cortex. Yet, the proteomic analysis identified the enrichment of DE-proteins in the cerebrum of middle-aged mice in the “regulation of neuronal synaptic plasticity” and “synapse” pathways. Collectively, these results indicate a reprogramming of synaptic health-associated gene expression profiles, though these changes appear insufficient to disrupt synaptic homeostasis in the middle-aged brain. The effects of CX3CL1/CX3CR1 signaling on synapses appear to be life stage- and context-dependent. During CNS development, this signaling pathway coordinates microglial recruitment and precise positioning to mediate synaptic pruning and maturation. This is supported by the observation that CX3CR1-deficient mice exhibit increased synaptic density during early postnatal development compared to wild-type controls (Mecca et al., 2018). In adulthood, CX3CL1/CX3CR1 signaling plays a key role in neurogenesis by regulating microglia-neuron crosstalk through cytokine and neurotrophic factor release, thereby modulating synaptic transmission and plasticity (Camacho-Hernández and Peña-Ortega, 2023). Our study revealed that CX3CR1 knockout reduced synaptophysin expression in the hippocampus and frontal cortex of middle-aged mice while increasing hippocampal levels of TNF-α and IL-1α. These findings suggest that CX3CL1/CX3CR1 signaling helps maintain synaptic homeostasis, probably in part through modulating microglial cytokine production. This conclusion aligns with previous reports demonstrating that CX3CR1 deficiency promotes NF-κB p65 activation and elevates IL-1β expression, leading to impaired hippocampal neurogenesis (Rogers et al., 2011; Sellner et al., 2016).

This study acknowledges several limitations. Firstly, the proteomic analysis utilized whole cerebral tissue rather than separately examining the hippocampus and frontal cortex. Investigating distinct brain regions is essential, as recent single-cell RNA sequencing studies have revealed varying molecular fingerprints and functions of microglia and astrocytes across different regions during brain development and in pathological conditions (Lee et al., 2022; Tan et al., 2020). In accordance, our study identified elevated TNF-α and IL-1α expressions in the hippocampus of CX3CR1-deficient mice, while no significant effects were observed in the frontal cortex. Secondly, we performed proteomic profiling on proteins extracted from formalin-fixed tissue. Although the extraction method has been optimized for proteomic analysis (Davidson et al., 2023; Gustafsson et al., 2015), caution is warranted in interpreting results due to potential protein cross-linking, loss of protein integrity, and limited detection of low-abundance proteins. Lastly, while we measured the expression levels of synaptophysin and PSD95 to assess synaptic homeostasis, this approach may be insufficient. Integrating behavioral tests, electrophysiological recordings, and imaging techniques to assess neuronal and synaptic function and structure would provide a more comprehensive analysis of synaptic plasticity. Despite these limitations, our study is the first to demonstrate the critical role of CX3CR1 elevation in maintaining synaptic homeostasis in the middle-aged mouse brain. Middle age may represent a crucial period when the balance between the production and clearance of harmful substances, such as cellular debris and misfolded proteins, shifts toward an uncontrolled state in the CNS, a process likely influenced by CX3CR1 autoregulation through its modulation of microglial function. Further investigations are warranted to validate time-dependent changes in CX3CR1 across the mouse lifespan and to identify its association with age-related neurodegeneration.

## 5 Conclusion

Our study demonstrated that CX3CR1 expression was upregulated in the hippocampus and frontal cortex of middle-aged mice, correlating with increased levels of scavenger receptors and decreased levels of proinflammatory cytokines. Furthermore, our findings indicated that CX3CR1 deficiency led to downregulated scavenger receptors, upregulated proinflammatory cytokines, altered microglial and astrocyte morphology, and reduced synaptic density, which might contribute to the vulnerability of the aging brain. This study underscores the importance of CX3CR1 upregulation in modulating microglial function and maintaining synaptic homeostasis during middle age.

## Data Availability

The mass spectrometry proteomics data presented in this study are deposited in the PRIDE partner repository, accession number PXD058961.
